# Physical Properties and Consumer Evaluation of Cocoa Bean Shell-Functionalized Biscuits Adapted for Diabetic Consumers by the Replacement of Sucrose with Tagatose

**DOI:** 10.3390/foods9060814

**Published:** 2020-06-21

**Authors:** Olga Rojo-Poveda, Letricia Barbosa-Pereira, David Orden, Caroline Stévigny, Giuseppe Zeppa, Marta Bertolino

**Affiliations:** 1 Department of Agriculture, Forestry and Food Sciences (DISAFA), University of Turin, 10095 Grugliasco, Italy; letricia.barbosa.pereira@usc.es (L.B.-P.); marta.bertolino@unito.it (M.B.); 2 RD3 Department-Unit of Pharmacognosy, Bioanalysis and Drug Discovery, Faculty of Pharmacy, Université libre de Bruxelles, 1050 Brussels, Belgium; Caroline.Stevigny@ulb.be; 3 Department of Analytical Chemistry, Nutrition and Food Science, Faculty of Pharmacy, University of Santiago de Compostela, 15782 Santiago de Compostela, Spain; 4 Departamento de Física y Matemáticas, Universidad de Alcalá, 28805 Alcalá de Henares, Spain; david.orden@uah.es

**Keywords:** cocoa bean shell, cocoa by-product, functionalized biscuits, tagatose, fiber content

## Abstract

The cocoa bean shell (CBS), a by-product of the cocoa industry, has been reported to be rich in fiber and polyphenols, which could contribute to reducing the metabolism of sugars and glucose adsorption. The production of CBS-based biscuits in which sucrose is replaced with tagatose (a low-glycemic sugar with prebiotic properties), benefiting diabetic consumers, is proposed. Six prototype biscuits were produced using sucrose, tagatose, and CBS powder at 0%, 10%, and 20% as a wheat flour replacement. Biscuits were studied in terms of fiber content, and those with 10% and 20% CBS showed to contain 5.66% and 8.70–8.71% of total dietary fiber, respectively. Moreover, the physicochemical and structural properties of the biscuits were studied to evaluate their differences due to the use of sucrose and tagatose combined with CBS. Significant effects mainly caused by the reducing nature and lower solubility of tagatose with respect to sugar, and the water retention capacities of CBS were observed. Finally, the biscuits were evaluated by performing a consumer acceptance evaluation, and their perceptible sensorial differences were studied by performing a Napping^®^ sensory characterization. CBS-based biscuits represent an interesting possibility for cocoa by-product revalorization, although an optimized recipe is recommended, especially when employing tagatose.

## 1. Introduction

Nutraceuticals and functional foods have received increasing interest among consumers in the last several years. One of the main reasons for this is the introduction of the idea that the diet can promote health benefits, reducing the risk of several diseases, such as cardiovascular affections, diabetes, obesity, cancer, osteoporosis, and arthritis, among others. Furthermore, emerging healthy eating trends and concerns, which are becoming increasingly prevalent in our societies in recent years, have given force to these new concepts. The term nutraceutical, first described in 1989, unites nutrition and pharmaceuticals, and it is defined as any substance that is considered a food or part of one and is able to provide medical or health benefits, including disease prevention and treatment, and that can be presented in different forms, from dietary supplement capsules to different processed foods, consumed mainly for health reasons [1]. On the other hand, functional foods could be considered a type of nutraceutical and are described as fortified foods with dietary components that can provide health benefits beyond basic nutrition [1]. Notably, polyphenols are one of the compound groups most commonly characterized as a nutraceutical. Polyphenols are non-nutritive microconstituents present in vegetable and cereal products, are usually employed in functional foods, providing them with special characteristics such as antioxidant, antidiabetic, or anticarcinogenic activities, among others [2]. Another compound responsible for imparting functional characteristics to foods is dietary fiber, whose consumption has been associated with the reduced incidence of disorders and diseases, such as chronic bowel disorders, obesity, diabetes, cardiovascular diseases, and cancer, among others [3]. Similar to polyphenols, dietary fiber does not have nutritional properties since it cannot be digested by the human organism, but it contributes to proper intestinal transit while retaining compounds and slowing glucose, lipid, sterol, and bile acid absorption [4,5]. For these reasons, dietary fiber plays a crucial role in the diet, and the European Food Safety Authority (EFSA) recommends a daily intake of 25 g of fiber for adults [6].

In particular, great attention has been given to vegetable and fruit by-products as nutraceuticals or ingredients for functional foods because of their content in interesting compounds, combined with the aim to create revalorization strategies for food industry wastes. In the manufacture of cocoa, one of the main by-products is the cocoa bean shell (CBS), which is the external tegument covering the cocoa bean. CBS constitutes 10–17% of the total cocoa bean weight and is normally discarded after its separation from roasted cocoa beans, entailing economic and environmental issues since more than 700,000 tons of CBS is estimated to be produced yearly around the world [7]. Nevertheless, CBS has demonstrated to be an interesting food ingredient since it has a similar nutritional composition to that of cocoa nibs (except for the fat content, which is substituted by fiber in the case of CBS) [7] and a similar aromatic profile to that of cocoa powder [8,9]. However, what attracts more attention in scientific research and adds value to this cocoa by-product is its content in dietary fiber, which ranges from 39.3% to 66.3%, and polyphenols, mainly flavanols [7]. Indeed, several researchers have studied the possibilities of its utilization as a fortifying ingredient in different foods, mainly baked goods such as functional cakes [10], muffins [11], bread [12], and biscuits [13]. Both CBS dietary fiber and polyphenols have been shown to give special characteristics to CBS regarding human health, which means that CBS may be considered a potential nutraceutical. In particular, dietary fiber and polyphenols in CBS have demonstrated different biofunctional potentials, such as antibacterial and antiviral properties, benefits to the cardiovascular system, anticarcinogenic effects, antidiabetic properties, anti-inflammatory effects, and even neuroprotective properties [7]. In this work, a special focus is given to the CBS antidiabetic aspect, which, in the case of CBS polyphenols, has been demonstrated to be caused by insulin secretion regulation, pancreatic cell protection, inhibition of glucose degradation enzymes, and insulin sensitivity enhancement, among other effects [7]. Indeed, some researchers have taken advantage of this fact for the development of functional beverages with antidiabetic properties [14,15]. In the case of CBS dietary fiber, some studies have reported that its antidiabetic properties are due to its capacity to absorb glucose during gastrointestinal digestion, retarding its diffusion and absorption through the intestinal wall [7]. This antidiabetic potential of CBS is of great importance, considering that the World Health Organization estimated that 422 million people worldwide aged over 18 years were living with diabetes in 2014 and that this number will be doubled by 2030 [16]. Therefore, increasing interest has appeared in the last several years for alternative ways to treat or prevent diabetes, mainly through the diet, by developing new foods adapted for diabetic consumers.

**Figure 1:**
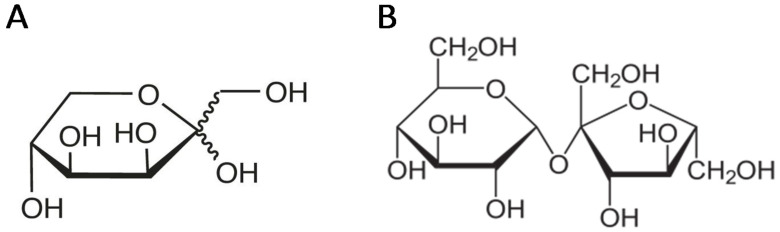
Chemical structures of tagatose (d-tagatopyranoside) (**A**) [19] and sucrose (α-d-glucopyranosyl-(1→2)-β-d-fructofuranoside) (**B**) [26].

On the other hand, for similar reasons, increasing interest has appeared for alternative low-glycemic sweeteners in recent years. One of these alternative sweeteners is tagatose, which is a natural hexoketose (a d-galactose isomer) considered a rare sugar because of its limited occurrence in nature (Figure 1A) [17]. It can be obtained by both chemical and biological processes: the former one is based on d-galactose isomerization catalyzed by calcium or sodium hydroxide, and the latter one involves galactitol dehydrogenase, l-arabinose isomerase, different bacteria species capable of acting as a biocatalyst, or production by fungal conversion of d-psicose into d-tagatose, among other biological converters [17,18]. Although it has up to 92% of the common sucrose sweetness and a similar taste without a cooling effect or aftertaste, tagatose presents a low caloric value (1.5–3 kcal/g) and is tooth friendly, without the laxative effects produced by other reduced-calorie sweeteners such as polyols [17,18,19,20]. Tagatose has been considered a nutraceutical compound; it shows beneficial effects on obesity control, pregnancy and fetal development, and blood factor regulation and has anti-aging properties, antioxidant properties, and, especially, prebiotic and antidiabetic properties [17]. Its prebiotic properties are due to the fact that tagatose is a mal-absorbing sugar because of its spatial configuration at the C-4 position, and therefore, only 20–25% of it is absorbed in the small intestine, while the rest reaches the large intestine and is subjected to fermentation by the gut microflora. This fermentation leads to the production of several short-chain fatty acids (SCFA), among which butyrate plays an important role since it is the major fuel for colonic epithelial cells, contributing to their development, acting as an anti-inflammatory agent, and helping in the prevention of colon cancer [17]. On the other hand, its antidiabetic properties are based on the fact that this rare sugar is able to avoid high peaks in the postprandial glycemic index, blunting the rise of blood glucose levels, with no influence on the insulin levels thanks to its particular metabolism in the liver and its inhibition of carbohydrate-digesting enzymes in the small intestine, such as sucrase and maltase [17]. Tagatose was declared a ‘generally recognized as safe’ (GRAS) product by the United States Food and Drug Administration (FDA) in 2001, which authorized its use at up to 30% *w*/*w* in baked goods [21], and its consumption as a novel food in the European Union was authorized by EFSA in June 2005 [20]. Indeed, since that moment, several researchers have studied the possibilities of its inclusion in functional foods to replace common sucrose, mainly in baked goods [22,23]. However, when compared with common sucrose (Figure 1B), tagatose has some different properties that could influence foods from a technological and sensory point of view. Among other differences, tagatose is a reducing sugar, while sucrose is not, and tagatose has a lower solubility when compared with sucrose (160 g/100 mL, 58% at 20 °C and 200 g/100 mL, 67% at 21 °C, respectively) [19,24,25], and it is also less hygroscopic than the latter [17,25].

As mentioned above, several researchers have studied the possibilities of using CBS and tagatose separately for the development of functional foods. However, to our knowledge, there are no studies in the literature proposing the use of these two biofunctional ingredients together. The aim of this work is, therefore, to develop CBS-functionalized biscuits using tagatose as a sweetener agent and, in this way, getting the advantage of the fiber content and antidiabetic capacities of CBS, together with the sweetener properties and beneficial effects of tagatose. To this end, the combined effects on the physicochemical and sensory properties of the developed biscuits were studied by adding different percentages of CBS flour in place of wheat flour (10% and 20%) and by substituting sucrose with tagatose. For comparison purposes, a sucrose control group and biscuits with no CBS substitution were also produced and tested.

## 2. Materials and Methods

### 2.1. Chemicals

Ethanol (99%), acetone (≥99%), sulfuric acid (96%), oxygen peroxide (50%), and Nessler’s reagent were obtained from VWR chemicals (Leuven, Belgium). Celite, sodium hydroxide (1N solution), and hydrochloric acid (≥32%) were acquired from Sigma-Aldrich (Steinheim, Germany). MES hydrate (>99%) and ammonium sulfate were provided by Alfa Aesar (Kandel, Germany). TRIS (>99.8%) was obtained from Acros Organics (Geel, Belgium). Petroleum ether (60‒80 °C) was supplied by LAB-SCAN analytical sciences (Gliwice, Poland). Amylase thermostable (3000 U/mL), protease (9 tyrosine equivalent units/mg), and amyloglucosidase (3260 U/mL), suitable for AOAC International total dietary fiber and starch analytical procedures, were obtained from Megazyme (Te Huissen, The Netherlands). Ultrapure water was prepared in a Simplicity UV water purification system (Millipore, Molsheim, France).

### 2.2. Samples

Roasted CBS from São Tomé cocoa beans (Forastero variety) was kindly provided by Pastiglie Leone S.r.l. (Turin, Italy). CBS was first ground to a grain size lower than 500 μm using a BA200N vibrating sieve (CISA, Barcelona, Spain). Then, it was micronized in a ball mill until obtaining CBS powder with a particle size below 20 μm, which was confirmed by measurement with a PSA 990 particle size analyzer (Anton-Paar GmbH, Graz, Austria). The characterization of the employed roasted CBS was perform on a previous work [14] with the following results: protein, 20.9 g; fat, 2.3 g; carbohydrates, 7.85 g; dietary fiber, 55.1 g; water, 5.9 g; and ash, 7.9 g. Tagatose was supplied by NuNaturals (Eugene, OR, USA).

### 2.3. Formulation of the Biscuits

Six different types of biscuits were produced using three percentages of wheat flour substitution by CBS powder (0%, 10%, and 20%) and two different types of sugar (sucrose and tagatose). The six biscuit types were coded: 0S (0% CBS and sucrose), 0T (0% CBS and tagatose), 10S (10% CBS and sucrose), 10T (10% CBS and tagatose), 20S (20% CBS and sucrose), and 20T (20% CBS and tagatose). Biscuits were formulated and baked according to the official method AACC 10-53.01 [27] with slight modifications, which comprises the use of butter instead of all-purpose shortening and the elimination of nonfat dry milk and high-fructose corn syrup as ingredients. The ingredient quantities employed are shown in Table 1.

All ingredients, except CBS and tagatose, were acquired from local markets. Water was added to the dough in variable quantities, depending on the CBS powder quantity that was added in place of wheat flour, in order to counterbalance the lower moisture provided by the former in comparison with the latter. All ingredients were homogeneously mixed in a KitchenAid^®^ dough beater (Whirpool Corporation, Benton Harbor, MI, USA). Butter was first whisked alone for one minute. After that, sugar or tagatose, salt, baking powder, and cocoa powder were added and mixed for three minutes. Then, water was added and mixed for another minute. Finally, wheat and CBS flours were added, and the dough was further mixed for three minutes. The obtained dough was gauged to obtain a 7-mm-thick dough sheet and cut with a circular mold to obtain 6-cm-width biscuits that were baked in a ventilated OLIS oven 044-054S (Ali Group, Milan, Italy) at 180 °C for 15 min. The different quantities of added CBS in place of wheat flour accounted for 0%, 5.28%, and 10.56% (*w*/*w*) of the total final biscuit weight for the 0%, 10%, and 20% substitutions, respectively. For the analyses that required a powdered sample, the biscuit samples were converted into 500‒1000-μm particle size powder by using an Ultracentrifugal Mill ZM 200 (Retsch GmbH, Haan, Germany).

### 2.4. Analytical Procedures

#### 2.4.1. Total Dietary Fiber Determination

Total dietary fiber content was determined by implementing the gravimetric AOAC official method 991.43 [28] on a Fibertec system E composed of a 1023 filtration module and a 1024 shaking water bath (Foss Tecator, Hilleroed, Denmark).

Before dietary fiber analysis, dried CBS flour and powdered biscuit samples were defatted and desugared as indicated by the official method. For the defatting phase, samples were extracted three times with 25 mL of petroleum ether per gram of sample for five minutes with strong agitation by a Reax 2 overhead shaker (Heidolph Instruments, Schwabach, Germany). Sugar was extracted three times with 85% ethanol for five minutes on the overhead shaker. Samples were dried overnight in a Venti-Line ventilated oven (VWR, Leuven, Belgium) after each phase and before dietary fiber determination.

For sample digestion, 40 mL of 0.05M MES-TRIS buffer pH 8.2 was added to 1.0000 g of dried, defatted, and desugared CBS or biscuit sample, and the mixture was stirred until the sample was dispersed. Analyses were performed in quadruplicate, and blanks were prepared and analyzed together with the samples during the whole process. The solutions were incubated with 50 μL of thermostable amylase, covered with aluminum foil for 15 min in a bath at 100 °C, and left to cool to 60 °C. Any ring or gels were scraped before adding 100 μL of protease (50 mg/mL) and incubating while covered with aluminum with agitation at 60 °C for 30 min. After that, 5 mL of 0.561N HCl was added while stirring, and pH was adjusted with a PP-15 pH-meter (Sartorius, Goettingen, Germany) at 60 °C to 4.0–4.7 by adding 1N NaOH or 1N HCl. Finally, 30 μL of amyloglucosidase was added, and the solution was further incubated with agitation for 30 min at 60 °C. Then, 200 ml of 95% ethanol at 60 °C was added, and the solution was left to precipitate for 1 h while covered with aluminum foil.

Approximately one gram of celite was previously added and washed with 15 mL of 78% ethanol on previously weighed Fibertec adapted filter crucibles and dried overnight. The resulting digestate was filtered through the crucibles and washed with 78% ethanol. Additional washes were done with 15 mL of 78% ethanol, 95% ethanol, and acetone (two times each), and crucibles were dried overnight to obtain the digestate residue weight. Two replicates of the obtained digestate, composed of fiber, proteins, and inorganic residues, were incinerated for 5 h at 525 °C on a muffle furnace (Nabertherm, Lilienthal/Bremen, Germany) for ash calculation.

The two remaining replicates were used for protein determination. To do this, a Digesdahl system (Hach Company, Loveland, CO, USA) was employed. The crucible content (celite + digestate residue) was transferred to a beaker and stirred with 5 mL of 50% H_2_SO_4_ for 30 min. The solution was transferred to the Digesdahl digestion flask, washed twice with 500 μL of 50% H_2_SO_4_, and heated inside the Digesdahl system at 440 °C until water evaporation (release of white smoke). Four minutes after this moment, 10 mL of 50% H_2_O_2_ was added at a 3 mL/min flux employing a capillary funnel and heating for another two minutes. After complete cooling, ultrapure water was added to the solution up to 100 mL. The ammonium sulfate formed from the protein nitrogen after the complete destruction of the organic matrix was then monitored by UV spectrophotometry with a Genesys 10UV UV–Vis spectrophotometer (Thermo Spectronic, Rochester, NY, USA). The obtained solutions from the protein digestion were further diluted 50 times, and 1 mL of Nessler reagent was added to 25 mL of solution. Absorbance was measured at 420 nm, and a standard curve made with (NH_4_)_2_SO_4_ corresponding quantities for 0–80 μg of nitrogen was employed to estimate the nitrogen content. Protein content was obtained by multiplying the nitrogen content by a factor of 6.25.

The total dietary fiber content was calculated as follows:

$$\mathrm{DF}\;\left(g/100g\right)=\left\{\left[\left(R_{1}+R_{2}+R_{3}+R_{4}\right)/4\right]-P-A-B\right\}/\left[(S_{1}+S_{2}+S_{3}+S_{4}\right)/4]\times 100$$

where R_1_–R_4_ are the residue weights for sample replicates (mg), P is the average protein weight of the final digestate residue (mg), A is the average ash weight of the final digestate residue (mg), B is the blank residue weight (mg), and S_1_–S_4_ are the initial weights for sample replicates (mg). Blank residue weight was calculated as follows:

$$B=\left[\left({\mathrm{BR}}_{1}+{\mathrm{BR}}_{2}+{\mathrm{BR}}_{3}+{\mathrm{BR}}_{4}\right)/4\right]-P_{B}-A_{B}$$

where BR_1_–BR_4_ are the residue weights for blank replicates (mg), P is the average protein weight of the blanks (mg), and A is the average ash weight of the blanks (mg).

#### 2.4.2. Physicochemical Analyses

A MAC210/NH thermo-moisture analyzer (Radwag, Radom, Poland) was used for moisture calculations, for which 5 g of powdered biscuits was used. For water activity determination, 2 g of powdered biscuits was used on an AquaLab PRE water activity meter (Decagon devices, Pullman, WA, USA).

Color analyses were conducted as in [14], using powdered biscuit samples on a CM-5 spectrocolorimeter (Konica Minolta, Tokyo, Japan) on transmittance and SCE (Specular Component Excluded) mode. The color space parameters L*, a*, and b* (CIELAB values) were used to measure the colorimetric characteristics.

#### 2.4.3. Structural Analyses

Weight loss was determined by weighing the biscuits before and after baking, and it was calculated as the percentage of the initial uncooked biscuit weight that was lost after the baking process. A total of 12 samples per biscuit group were used for the weight loss calculation.

The width and thickness measurements and the spread calculation were taken following the AACC 10-50.05 method [29]. The width and the thickness were measured for 12 baked samples per biscuit group and used to calculate the spread parameter. The spread was calculated as follows: $\mathrm{Spread}=\frac{\mathrm{width}}{\mathrm{thickness}}\times \mathrm{conversion}\;\mathrm{factor}\times 10$, where the conversion factor is a parameter dependent on the atmospheric pressure and the height level above the sea on the day in the laboratory where the analyses were conducted, and it is reported in the mentioned method.

The hardness of the biscuit samples was measured on a TAXT2i Texture Analyzer^®^ (Stable Micro Systems, Godalming, UK) equipped with a 25-kg load cell. An HDP-BS cutting blade was used for the analysis at a speed of 1 mm/s. For each biscuit prototype, a set of 12 biscuits was used for measuring this parameter. Version 2.54 of the Texture Expert Exceed software package for Windows (Stable Micro Systems, UK) was used for data acquisition, and the results were expressed in N as the maximum force needed for breaking the biscuit.

### 2.5. Sensory Analyses

#### 2.5.1. Consumer Evaluation

A consumer acceptance evaluation was performed as in [14] with a panel of 25 untrained tasters, to whom biscuits were given in random order. Tasters were asked to evaluate different parameters, such as appearance, odor, taste, flavor, texture, overall liking, and purchase predisposition on a 9-point hedonic scale (1 = extremely dislike, 9 = extremely like) [30]. Water was provided for mouth rinsing between sample tastings. Tests were carried out in an adapted air-conditioned room that was equipped with white light and was approximately 21 °C.

#### 2.5.2. Napping^®^ Sensory Analysis

For the Napping holistic sensory analysis [31], projective mapping positioning was performed by 12 tasters during two sensory sessions. The six different biscuits were given simultaneously to the tasters, who were asked to taste them in different random orders. Tasters were then asked to place the samples on a 40 × 60 cm tablecloth according to their similarities (the more similar the biscuits were perceived, the closer they should be positioned), without specific direction for sample positioning. Next, tasters were asked to freely attribute some descriptive terms to the samples and to write them down next to the samples positioned on the tablecloth, according to the Ultra-Flash Profiling (UFP) approach [32].

Measures were taken for each sample positioned in the sensory space, represented by the x- and y-axes, where the left lower corner was considered the axis origin point (0,0). The different UPF terms were then collected, and their frequencies were computed when repeated. Additionally, UPF terms with similar meanings were grouped as shown in Table 5. All the terms were collected in a contingency table, which was added as supplementary information to the sensory space position of each sample for further statistical analysis.

### 2.6. Statistical Analysis

The results of fiber, physicochemical, and structural analyses were subjected to one-way analysis of variance (ANOVA) with Duncan’s post hoc test at a 95% confidence level in the SPSS Statistics 25 software (IBM-SPSS Inc., Chicago, IL, USA).

Values obtained by the consumer acceptance test were analyzed by the Kruskal–Wallis test (test H) in the STATISTICA software for Windows, version 13.3 (StatSoft Inc., Tulsa, OK, USA).

Data obtained from the Napping^®^ sensory analysis (positions in the sensory space) were treated through a geometric analysis using SensoGraph in order to obtain proximity graphs from the Napping^®^ data. Python implementation [33] of the SensoGraph method proposed in [34] was used to analyze the (x,y) coordinates provided by the tasters. This method comprises three steps: first, a clustering technique from Computational Geometry called the Gabriel graph is applied to each tablecloth so that only pairs of samples without third samples nearby are connected. Second, a global similarity matrix is created, whose i,j entry is 1 if samples i–j are connected and 0 otherwise. Third, a graph-drawing algorithm uses that matrix to provide the 2D positioning of the samples.

## 3. Results and Discussion

### 3.1. Total Dietary Fiber Content

For each type of biscuit, total dietary fiber content (TDF) values are reported in Table 2. The obtained value for the CBS powder was 61.87 ± 1.50 g of TDF per 100 g of dried CBS, which is in accordance with the gravimetrically obtained TDF values reported in the literature [7].

Statistically similar values were found when comparing biscuits with the same CBS content but different sugar groups, proving that the employed sugar had absolutely no influence on this parameter. For the control biscuits, values of 2.48 g and 2.79 g TDF/100 g of dried product were found for S0 and T0, respectively. These values are in accordance with the work of Kārkliņa et al. [13], who obtained a TDF value of 2.9 g TDF/100 g for a sugar control biscuit with a similar composition to the ones employed in this study. Increasing CBS content significantly increased the TDF in both sugar biscuit groups to an expected extent when taking into account the CBS quantity added and the fact that this ingredient possesses 61.87% TDF. In this way, we obtained values of 5.66 g TDF/100 g for both S10 and T10, in which the CBS final content of the biscuit was 5.28 g of CBS/100 g of biscuit (10% wheat flour substitution). Again, these values were in accordance with the previously mentioned research work, in which 4.22 g TDF/100 g was found for biscuits with a CBS final content of 4.33 g per 100 g of biscuit (10% wheat flour substitution) [13]. On the other hand, values of 8.70 and 8.71 g TDF/100 g were found for S20 and T20, respectively, which are the biscuits in which 20% of the wheat flour was substituted with CBS powder.

In view of these results and according to the European Union regulation for nutrition and health claims for food [35], both the sucrose and tagatose biscuits in which 10% of the wheat flour was substituted with CBS could be claimed as a ‘source of fiber’ since both S10 and T10 contained more than 3 g of fiber per 100 g of product, which is the minimum necessary for this claim. On the other hand, and according to the same regulation, both S20 and T20 could be commercially claimed as ‘high-fiber’ biscuits since both surpass a quantity of 6 g of fiber per 100 g of product, which is the minimum necessary for this other claim.

### 3.2. Physicochemical Characterization

Moisture, water activity, and CIELAB parameters for color measurement of the biscuits are compiled in Table 2.

Moisture and water activity (a_w_) are two important parameters to be monitored for the storage stability of biscuits since these factors can influence their resistance to microbes and their rheological properties [36]. The results for both parameters were found to be significantly different for all six types of biscuits. Thus, differences in these parameters were found for both variables of ‘different CBS percentages within the same sugar group’ and ‘different sugars within the same CBS percentage’. Moisture and a_w_ had proportionally correlated results, which was expected since a_w_ is known to be significantly influenced by moisture. For both sugars, humidity and a_w_ considerably increased when increasing the percentage of CBS added in place of the wheat flour. This effect could be explained by the higher dietary fiber content provided by CBS in comparison with that in wheat flour, which provides a higher water retention capacity, as described by Martínez-Cervera et al. [11]. On the other hand, if the comparison is made between the different employed sugars, it can be observed that, for the same CBS percentage, the tagatose biscuits, in all cases, had higher moisture and a_w_ values than the biscuits made with sucrose. Being less soluble than sucrose, tagatose can allow for greater re-crystallization after baking, which results in water release and, thus, higher moisture and a_w_ values [22,37]. Nevertheless, the water activity values of all the studied biscuits were below the 0.60 threshold under which they can be considered microbiologically stable [36].

**Figure 2:**
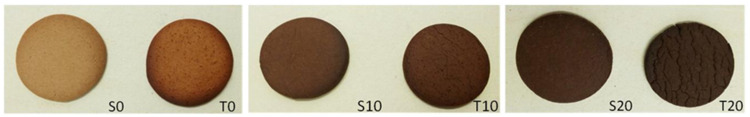
Visual comparison between the six different biscuit types for color-contrasting purposes. From left to right: S0 (sucrose control biscuit with 0% of wheat flour substituted with CBS), T0 (tagatose control biscuit with 0% of wheat flour substituted with CBS), S10 (biscuit with sucrose and 10% of wheat flour substituted with CBS), T10 (biscuit with tagatose and 10% of wheat flour substituted with CBS), S20 (biscuit with sucrose and 20% of wheat flour substituted with CBS), and T20 (biscuit with tagatose and 20% of wheat flour substituted with CBS).

For contrast purposes, Figure 2 shows the six different biscuit types for a visual comparison. The chromatic characteristics of the biscuits were studied through the CIELAB color space, composed of L*, which measures the lightness of the samples from 0 (black) to 100 (white); a*, which measures the degree of redness (when positive) or greenness (when negative); and b*, which indicates the yellowness (when positive) or blueness (when negative) of the sample color [14]. When looking into the obtained CIELAB values for all six biscuit types, the most relevant parameter was L*. This parameter was first observed to significantly decrease when increasing %CBS within both sugar groups, as the biscuits became darker because of the brown color provided by the CBS ingredient. Besides this, significant differences can be observed in the L* parameter of both control biscuits S0 and T0, where the CBS had no influence. T0 had a significantly lower L* value than that of S0, with values of 44.78 and 50.78, respectively. This fact is explained by the reducing nature of tagatose in contrast to sucrose, which is not a reducing sugar. This allows for an extra browning of the tagatose biscuit surface during baking (observed in Figure 2 for T0) since Maillard browning reactions may occur between the reducing sugar (tagatose) and the proteins contained in the biscuit dough, as reported by Struck et al. [38]. Both the a* and b* parameters significantly decreased for both sugar groups when increasing the CBS content, meaning that the biscuits became less red and less yellow. In this case, it is important to remark that, regarding a* and b*, significant differences were found between sugars in the control biscuits and in those with 10% of the wheat flour substituted by CBS, with these values higher for the tagatose group in both cases. However, both biscuits with the higher percentage of CBS (S20 and T20) had statistically similar values of the three chromatic parameters, meaning that at this percentage of wheat flour substituted with CBS, the employed sugar no longer had an influence on the color.

Concurrently, for comparison purposes in terms of color, the ΔE parameter was calculated using the CIELAB parameters. This parameter is calculated according to the equation $\Delta E_{1-2}=\sqrt{{\left({L*}_{1}-{L*}_{2}\right)}^{2}+{\left({a*}_{1}-{a*}_{2}\right)}^{2}+{\left({b*}_{1}-{b*}_{2}\right)}^{2}}$, and it indicates whether the differences in color between two different samples are perceivable by the human eye (perceivable when ΔE ≥ 2.5) [39]. In the case of the studied biscuits, when comparing biscuits with the same CBS percentage, ΔE values were higher than 2.5 for the comparison S0−T0 and S10−T10 (ΔE_S0−T0_ = 5.22 and ΔE_S10−T10_ = 10.36), while ΔE resulting from the comparison between S20 and T20 was ΔE_S20−T20_ = 1.94. This means that, as perceived by the human eye, all the biscuits were different in color except for the ones with the higher percentage of CBS; the similarities between them, without regard to the employed sugar, were previously observed with the significant similarities in the CIELAB parameters, as observed in Figure 2.

### 3.3. Structural Characterization

The weight loss after baking, width, thickness, spread, and hardness of the biscuits were measured for structural characterization and are reported in Table 3. The influence of the biscuit ingredients’ nature on the structural and texture parameters of biscuits is well known. As reported by several authors, these characteristics depend on, among other parameters, the sugar nature and its degree of solubility, the protein content, and the amount of water available for gluten development and the relative extent of the creation of a gluten network [40].

Regarding weight loss, no significant differences were found among the different wheat flour substitutions with CBS for the same sugar group. However, a slight increase in weight loss was observed when increasing the CBS content for sucrose biscuits, and the opposite effect was observed for the tagatose biscuit group. This could be due to the lower solubility of tagatose during baking, which leaves more free water that is absorbable by the CBS fiber. This phenomenon is also confirmed by the humidity data (see Table 2).

The width parameter or biscuit diameter showed significant differences between both control biscuits without the CBS addition, with values of 6.51 and 6.12 cm in width for S0 and T0, respectively. The significantly lower width of the tagatose control biscuit was probably due to the lower water-binding capacity of tagatose compared with sucrose, which allows less spreading to occur because a lower amount of syrup is produced during baking, as reported by Taylor et al. [22], who obtained similar results when replacing sucrose with tagatose in cookies. However, when considering biscuits in which a percentage of wheat flour has been substituted by CBS, this effect was no longer observed, and no significant differences were observed between either the different sugars or different CBS additions. In these four cases, the effect of the incorporation of the CBS fiber prevailed over the difference between sugars for S10, S20, T10, and T20 biscuits. A similar slight decrease in the biscuit width was therefore observed when compared with the control biscuits as a result of the water absorption by the added CBS fibers [41].

The thickness or biscuit height results of the different biscuits followed a similar trend to that of the width parameter. No significant differences were found between the different CBS substitutions within the sugar groups or between both sugar groups, although a slight increase in this parameter was observed (mostly for the sucrose biscuits) when the CBS content was increased. This slight increase was probably due to higher gluten development because of a higher amount of water available. However, the sucrose control biscuit S0 presented a thickness value of 0.86 cm, which is significantly lower than the 0.92-cm thickness of the tagatose control biscuit T0. This difference can again be attributed to the lower hygroscopy of tagatose in comparison with sucrose, which, in this case, leaves more water available for the gluten proteins to create a network and to develop in height [22,40].

The biscuit spread parameter is the result of the adjusted ratio of the width and the thickness and, therefore, represents a combination of the different sugar and CBS content effects observed above. When comparing sugar groups, significant differences were only observed between the control biscuits with no CBS fortification. The sucrose control biscuit (S0) showed a 75.85-spread value, significantly higher than the 66.52-spread value of the tagatose biscuit (T0). As mentioned above, this result comes from the combination of the lower solubility of tagatose in comparison with sucrose (lower width) and its greater thickness due to the allowance for more gluten development. When observing the effect of the different CBS percentages on the spread value, significant differences were found within the sucrose biscuit group. The spread of the control biscuit S0 was found to be significantly higher than those of the CBS-fortified biscuits S10 and S20. In this case, a decrease in the spread was observed because the gluten network was diluted by the fiber addition. As described by Ktenioudaki and Gallagher [41], a higher fiber content absorbs some of the water, which is then no longer available for the gluten network development, which translates into a lower biscuit spread. In general, the inclusion of CBS in the biscuit formulation resulted in less spread and more compacted biscuits due to the increase in fiber content, which is in accordance with the results observed by Collar et al. [12] for CBS soluble fiber-fortified bread.

The hardness parameter represents the force required by the cutting probe to penetrate the biscuit and cut it into halves. It could, therefore, be understood as the necessary force to be employed for biting the biscuit. In the CBS functional biscuits, this parameter will be influenced by the interaction between the different ingredients. The sugar type can also influence this parameter since sugars may crystallize, which increases the biscuit hardness [22]. The obtained results for the hardness parameter showed that sucrose biscuits were significantly crispier than tagatose biscuits. This can be explained by the fact that sucrose possesses a higher solubility compared with tagatose, yielding a weaker gluten network, less sugar crystallization after the cooling of the baked biscuits, and, therefore, crispier biscuits, as described by Garcia-Serna et al. [42] and Mamat et al. [40]. When regarding the CBS content within the same sugar group, an increase in the hardness value was observed for the tagatose biscuits. These results were expected since increased CBS content leads to higher fiber content, which increases the biscuit hardness. CBS incorporation causes water absorption by the CBS dietary fiber, leaving less water for gluten development and, therefore, increasing hardness and losing crispiness since the biscuit mass does not retain gas (the biscuit surface breaks and cracks during baking, as observed for T20 in Figure 2) and does not expand as the control biscuit does [41]. The obtained tagatose biscuits were thus tough and not crumbly. Similar trends of the hardness parameter were observed in other research works in which CBS was added as a fat replacer for functional cake production [10] or for nutritionally fortified biscuit development [13]. However, in the sucrose biscuit group, the increasing CBS content did not lead to an increase in the hardness values, which were even lower for S10 and S20 than for S0. A hypothesis for these unexpected results might be that, in these biscuits, because sucrose is more soluble than tagatose, the sugar has already used the available water before CBS is added during the dough mixing, which does not leave much water available for its absorption by the CBS dietary fiber.

### 3.4. Sensory Analyses

#### 3.4.1. Hedonic Consumer Acceptance Evaluation

Results from the hedonic sheet compliance are shown in Table 4. Data are shown as the sums of the ranks obtained for each consumer evaluation parameter for each of the six biscuits. These values were subjected to a Kruskal–Wallis test to highlight differences in consumer acceptance between the six different biscuits.

Concerning the biscuit appearance, significant differences were observed between the sucrose and the tagatose groups, although tasters liked S20 and T10 to a similar extent. In general, sucrose biscuits were preferred, and lower values were found for T0 and T20. In the case of T0, this was probably due to the excessive browning color, caused by the Maillard reactions, that was observable in this biscuit (Figure 2, T0) and that gave a ‘burnt aspect’ to the biscuit. In the case of T20, the lower appearance preference was probably linked to the cracked aspect of the biscuit (Figure 2, T20), which broke during baking, possibly as a result of the absence of gluten network formation during the mixing process because of the fiber content, which led to greater gas release during the cooking phase.

As far as the odor is concerned, a similar trend was observed in which the sucrose biscuits were preferred, followed by T10, with T0 and T20 as the less liked biscuits. Again, this could be linked to the produced Maillard reactions, which were more prevalent in the tagatose biscuits and caused the development of some aromas that were not highly accepted.

The taste and flavor parameters showed similar results in terms of significant differences. The whole sucrose group was significantly more accepted than the whole tagatose biscuit group. Again, these values could be explained by the Maillard reactions that occurred to a greater extent in the tagatose biscuit group, developing a more bitter taste and undesired flavors.

Regarding texture, sucrose biscuits were again the most liked. Furthermore, a decrease in the acceptance was observed when increasing the CBS percentage in this group, presumably due to the greater moisture and water activity provided by CBS powder that made the biscuits denser to chew, as was also reported by Martínez-Cervera et al. [11]. Similar to the observations above, the rating values for the tagatose biscuits were much lower than the ones obtained for sucrose biscuits, decreasing with the addition of CBS. In this case, the texture parameter for T20 received the lowest acceptance value within the whole consumer evaluation. This fact is surely linked to the already reported high hardness of T20 and its high value of water activity (0.52) which, combined, resulted in tough and non-crumbly biscuits, since it has been reported that biscuits lose their crispiness at a_w_ values higher than 0.5 [43].

Finally, the overall liking and the purchase predisposition parameters were naturally linked and had similar results, in which the sucrose biscuits were significantly preferred to the tagatose biscuits, with S0 presenting the highest values and T20 the lowest.

Generally, sucrose biscuits were highly preferred over tagatose biscuits for all the different consumer acceptance parameters, although tagatose has been reported to not differ highly from sucrose in terms of taste [18,19]. For the sucrose group, it was observed that the addition of CBS caused a slight decrease in the acceptance values, although this decrease was not significant. However, it is interesting to remark that the opposite happened within the tagatose group, where the addition of CBS resulted in a rise in preference for almost all parameters (excepting texture) when considering T10 and, to a lower extent, T20.

#### 3.4.2. Napping^®^ Sensory Characterization

The Napping^®^ technique consists of a holistic approach (considering each sample as a whole), which has been shown to be very useful for sensory characterization, with advantages such as the possible discrimination among products [31]. In this work, the Napping^®^ results were analyzed through geometric analysis using SensoGraph [33], which allowed for better visualization of the obtained results.

Data from the Napping^®^ sensory characterization were obtained from the different positions that were given to the samples on the tablecloths. These positions were characterized by different UPF terms freely given by the tasters, which served as criteria for positioning samples in the sensory space and were, in some cases, grouped into categories for terms with similar meanings (Table 5).

**Figure 3:**
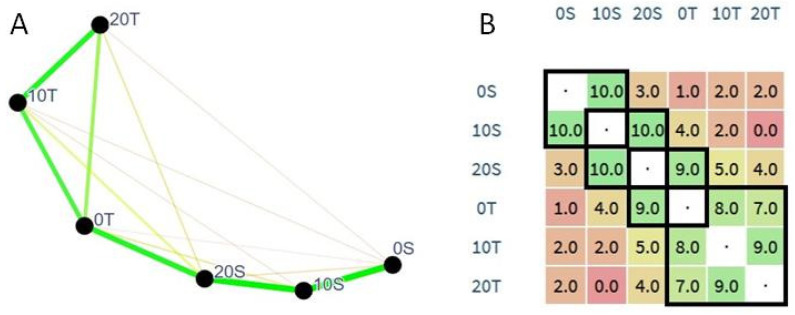
SensoGraph consensus plot of the samples tasted (**A**) and Global similarity matrix from the SensoGraph method, with groups highlighted as black squares (**B**).

The plots obtained from the SensoGraph geometric analysis of the Napping^®^ data are shown in Figure 3. Figure 3A shows the output provided by the SensoGraph method, as introduced in [34]. Samples appear in the diagram as black points labeled with the sample code. Samples that appear close to each other were perceived as more similar by the panel of tasters and vice versa. In addition, the segments between the samples represent the strength of the connections between samples using a two-fold codification: from red (weaker) to green (stronger) and from thin (weaker) to thick (stronger). In addition, Figure 3B shows the global similarity matrix obtained. For samples i and j, the position in the i-th row and the j-th column indicates the number of tasters for whom those samples became connected after the clustering process. The same color code explained above for the consensus plot is used here for the matrix.

A clear group of samples 0T-10T-20T appears on the consensus plots in Figure 3A, with strong mutual connections between these three samples (appearing as a triangle of thick green lines). The matrix shows that these three connections have similar and strong values (see the lower-right group in Figure 3B). Both facts show that the tasters identified a strong similarity between samples 0T-10T-20T, which is in accordance with the consumer acceptance evaluation results, in which the tagatose biscuits produced significantly similar results for almost all the hedonic parameters.

On the other hand, samples 0S-10S-20S form a line, with a strong connection for 0S-10S and a strong connection for 10S-20S, but a weak connection for 20S-0S. In other words, the tasters perceived sample 10S to be similar to both samples 0S and 20S, but they did not perceive samples 0S and 20S to be similar (see the remaining groups in Figure 3B).

Finally, Figure 3A shows that the triangle 0T-10T-20T and the path 0S-10S-20S are strongly connected through samples 0T and 20S, providing a kite-like configuration. This shows that the T- and S-samples were not perceived as completely independent, but that the tasters perceived a similarity between two samples of those types, namely, between 0T and 20S.

## 4. Conclusions

The addition of CBS powder in place of wheat flour led to the production of biscuits that meet the criteria for claiming them to be a ‘source of fiber’ and ‘high-fiber’ biscuits for the 10% and 20% wheat flour substitution, respectively. The various produced biscuits presented considerable differences at both technological and sensory levels. Both factors—the use of tagatose and increasing percentages of CBS—led to water retention, thus affecting the physical and structural properties of the biscuits. From a sensory point of view, sucrose biscuits were highly preferred over tagatose biscuits, which suggests important sensorial differences between the two sugars and the lack of appreciation by the tasters for the physical differences provided by tagatose utilization. However, it was observed that the addition of CBS enhanced the preferability of the tagatose biscuits, namely, those with 10% of wheat flour substituted with CBS flour (T10). The geometric analysis using SensoGraph of the Napping^®^ results showed a clear association of the biscuits depending on the employed sugar, although these groups were not perceived as completely independent.

The possibility of producing tagatose and CBS-based biscuits destined for diabetic consumers is confirmed by these results. However, an adapted recipe in which some of the tagatose is substituted by polyols is recommended in order to improve the tagatose biscuit characteristics (water adsorption, gluten network formation, taste, and aroma). Additionally, the specific aspects of the CBS-based biscuit functionality need to be assessed, which will be done in future work.

## Figures and Tables

**Table 1:** Quantities of ingredients employed for the biscuits’ preparation according to the official method AACC 10-53.01 [27], with slight modifications.

Ingredients	Quantities (g)		
	0% (Control Biscuits)	10% CBS Substitution	20% CBS Substitution
Wheat flour	225.0	202.5	180.0
Sucrose or tagatose	100.0	100.0	100.0
Butter (>82% fat)	90.0	90.0	90.0
CBS powder	0.0	22.5	45.0
Baking powder	7.0	7.0	7.0
Cocoa powder	3.0	3.0	3.0
NaCl	1.2	1.2	1.2
Water	51.0	55.0	69.0

**Table 2:** Total dietary fiber content, moisture, water activity, and CIELAB color values obtained for each biscuit and ANOVA among different percentages of CBS substitution within each sugar group and among both sugars for the same %CBS group.

Biscuit Sample	Sugar	CBS Percentage	Fiber			Moisture			a_w_			L*			a*			b*		
			(g/100 g Dried Product)			(%)														
S0	Sucrose	0	2.48	±	0.23 ^cA^	1.69	±	0.26 ^cB^	0.12	±	0.03 ^cB^	50.78	±	1.93 ^aA^	11.70	±	0.39 ^aB^	23.66	±	1.08 ^aB^
S10		10	5.66	±	0.56 ^bA^	3.21	±	0.14 ^bB^	0.25	±	0.00 ^bB^	33.56	±	0.38 ^bB^	12.04	±	0.29 ^aB^	17.10	±	0.48 ^bB^
S20		20	8.70	±	0.34 ^aA^	4.50	±	1.05 ^aB^	0.37	±	0.09 ^aB^	28.54	±	0.67 ^cA^	9.04	±	2.45 ^bA^	11.77	±	2.90 ^cA^
*Significance*			***			***			***			**			***			***		
T0	Tagatose	0	2.79	±	0.07 ^cA^	4.76	±	0.11 ^cA^	0.37	±	0.01 ^cA^	44.78	±	0.90 ^aB^	16.04	±	0.34 ^aA^	30.90	±	0.37 ^aA^
T10		10	5.66	±	0.58 ^bA^	5.69	±	0.46 ^bA^	0.43	±	0.02 ^bA^	35.49	±	1.66 ^bA^	13.76	±	0.32 ^bA^	21.63	±	1.20 ^bA^
T20		20	8.71	±	0.38 ^aA^	7.36	±	0.51 ^aA^	0.52	±	0.03 ^aA^	28.48	±	1.50 ^cA^	9.54	±	2.27 ^cA^	13.64	±	3.19 ^cA^
*Significance*			**			***			***			***			***			***		

Means followed by different lowercase superindexes indicate significant difference at *p* < 0.05 among CBS percentages within each sugar group; means followed by different uppercase superindexes indicate significant difference at *p* < 0.05 for different sugars within the same %CBS group; ** *p* < 0.01, *** *p* < 0.001; data are expressed as mean values (*n* ≥ 3) ± standard deviation.

**Table 3:** Weight loss after baking, width (diameter), thickness (height), spread, and hardness values obtained for each biscuit and ANOVA among different percentages of CBS substitution within each sugar group and among both sugars for the same %CBS group.

Biscuit Sample	Sugar	CBS Percentage	Weight Loss			Width			Thickness			Spread			Hardness		
			(%)			(cm)			(cm)						(N)		
S0	Sucrose	0	15.84	±	1.19 ^aA^	6.51	±	0.26 ^aA^	0.86	±	0.05 ^aB^	75.85	±	6.73 ^aA^	68.83	±	11.95 ^aA^
S10		10	16.08	±	0.48 ^aA^	5.93	±	0.10 ^bA^	0.91	±	0.08 ^aA^	65.28	±	6.53 ^bA^	53.92	±	6.46 ^bB^
S20		20	16.44	±	0.48 ^aA^	5.81	±	0.12 ^bA^	0.90	±	0.01 ^aA^	64.13	±	1.67 ^bA^	54.75	±	12.21 ^bB^
*Significance*			ns			***			ns			***			**		
T0	Tagatose	0	15.33	±	1.14 ^aA^	6.12	±	0.08 ^aB^	0.92	±	0.07 ^aA^	66.52	±	4.07 ^aB^	72.77	±	29.72 ^bA^
T10		10	14.25	±	0.46 ^aB^	5.88	±	0.07 ^bA^	0.89	±	0.10 ^aA^	66.47	±	7.61 ^aA^	88.96	±	19.39 ^abA^
T20		20	14.69	±	0.75 ^aA^	5.81	±	0.12 ^bA^	0.90	±	0.01 ^aA^	64.13	±	1.67 ^aA^	120.34	±	51.14 ^aA^
*Significance*			ns			***			ns			ns			*		

Means followed by different lowercase superindexes indicate significant difference at *p* < 0.05 among CBS percentages within each sugar group; means followed by different uppercase superindexes indicate significant difference at *p* < 0.05 for different sugars within the same %CBS group; * *p* < 0.05, ** *p* < 0.01, *** *p* < 0.001, ns = not significant; data are expressed as mean values (*n* ≥ 3) ± standard deviation.

**Table 4:** Consumer evaluation of the six biscuit types and results of the Kruskal–Wallis test (test H). Data are expressed as the sum of ranks of the results obtained from 25 tasters who filled out a 9-point hedonic scale (1 = extremely dislike, 9 = extremely like).

Biscuit Sample	Sugar	CBS Percentage	Appearance	Odor	Taste	Flavor	Texture	Overall Liking	Purchase Predisposition
S0	Sucrose	0	2602.0 ^a^	2890.0 ^a^	2843.5 ^a^	2877.5 ^a^	3057.0 ^a^	2961.5 ^a^	2992.5 ^a^
S10		10	2652.0 ^a^	2394.5 ^ab^	2737.5 ^a^	2716.5 ^a^	2754.0 ^a^	2773.5 ^a^	2823.0 ^a^
S20		20	1692.5 ^ab^	2175.0 ^abc^	2451.5 ^a^	2451.5 ^a^	2164.0 ^ab^	2339.0 ^a^	2365.0 ^a^
T0	Tagatose	0	1526.0 ^b^	994.0 ^d^	986.5 ^b^	1051.5 ^b^	1567.0 ^bc^	1146.5 ^b^	1009.0 ^b^
T10		10	1781.0 ^ab^	1502.0 ^bcd^	1226.0 ^b^	1225.5 ^b^	1078.5 ^c^	1149.5 ^b^	1199.5 ^b^
T20		20	921.5 ^b^	1369.5 ^cd^	1080.0 ^b^	1002.5 ^b^	704.5 ^c^	955.0 ^b^	936.0 ^b^
*Significance*			***	***	***	***	***	***	***

Means followed by different letters are significantly different at *p* < 0.05. Significance: *** *p* < 0.001.

**Table 5:** Grouped terms generated from the Ultra-Flash Profiling (UFP) during the Napping^®^ sensory analysis.

Friability	Hardness	Softness	Crunchy	Astringent	Sweet
Shortbread	Hard on the first try	Damp			Honey
Dry		Tender			Not sweet
Granulose		Undercooked			Jam
		Gummy			
		Soluble			
		Sticky			
**Bitter**	**Salty**	**Umami**	**Sour**	**Butter**	**Toasted**
Stale		Stock cube		Fermentation	Hazelnut
**Chocolate**	**Caramel**	**Burned**	**Cooked corn**	**Milk**	**Vegetal**
Unsweetened cocoa powder	Amaretto biscuit		Straw	Condensed milk	Wet grass
Chocolate milk			Puffed rice		
Sweet cocoa					
